# Accelerated delayed enhancement imaging of myocardial infarction with through-time radial GRAPPA

**DOI:** 10.1186/1532-429X-16-S1-W6

**Published:** 2014-01-16

**Authors:** Ozan Sayin, Haris Saybasili, Henry Halperin, Menekhem Zviman, Mark A Griswold, Nicole Seiberlich, Daniel A Herzka

**Affiliations:** 1 Department of Biomedical Engineering, Johns Hopkins School of Medicine, Baltimore, Maryland, USA; 2 Siemens Healthcare USA, Inc., Chicago, Illinois, USA; 3 Department of Radiology, Johns Hopkins Hospital, Baltimore, Maryland, USA; 4 Department of Biomedical Engineering, Case Western Reserve University, Cleveland, Ohio, USA

## Background

Delayed contrast enhancement (DCE) imaging is a well-established MRI technique for the evaluation of myocardial infarction (MI) and tissue viability [1]. Inversion-recovery (IR) is used to visualize the hyperenhanced regions of scar after injection of Gd-DTPA. Mid-diastolic segmented k-space coverage is typical, requiring several heartbeats to reconstruct an image [2]. More recently, Cartesian single-shot trueFISP imaging,[3] has achieved free-breathing acquisitions though spatial resolution is traded for imaging speed and multiple heartbeats may be needed to increase SNR through averaging [4]. Alternatively, radial imaging can achieve higher degrees of acceleration[5], and should produce sharper images in patients with high heart-rates and with shorter or non-existent quiescent periods. We demonstrate the feasibility of IR-DCE imaging with a rapid radial sequence accelerated using through-time radial GRAPPA with and without multi-heartbeat averaging.

## Methods

With ACUC approval, one swine with antero-septal MI was imaged at 1.5T (Avanto, Siemens Medical Systems, Erlangen, Germany). Images were acquired 15-25 min after administration of a single dose of gadopentetate dimeglumine. An 8-fold (R = 8) acceleration was achieved with radial sampling and 16 spokes were acquired per image, yielding a temporal footprint of 70 ms per image. Through-time radial GRAPPA [5] was used to reconstruct 1.56 × 1.56 mm^2 ^resolution images. For calibration of the weights, 80 fully-sampled (128 spokes) frames were acquired prior to infusion of contrast. An ECG-triggered IR-prepared gradient-echo sequence was utilized with FOV: 200 mm, 128 × 128 matrix, slice thickness: 7.0 mm, TR: 4.4 msec, BW: 797 Hz/Px, TI: 250-350 ms. Image reconstruction utilized a remote server with multi-CPUs and a GPU,6 enabling low-latency reconstructions with real-time inline display [6]. Short and long axis views were acquired. Images captured in single heartbeats were compared to images reconstructed after k-space averaging (8 heartbeats).

## Results

**Figure 1:**
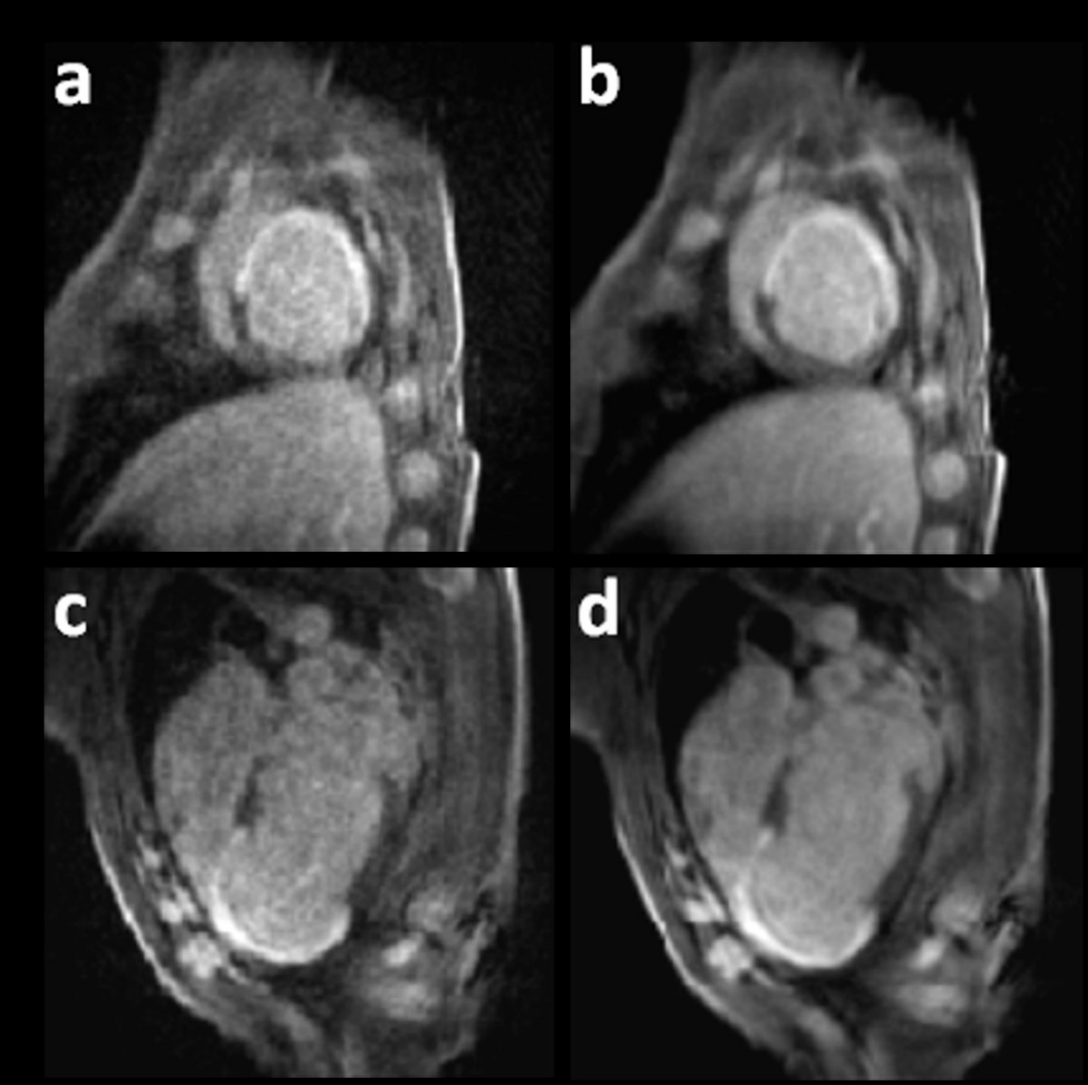
**DCE images of a swine with a chronic anteroseptal MI acquired with rGRAPPA (R = 8) and a temporal footprint of 70 ms**. Left: SAX and LAX view images captured in a single cardiac cycle are shown in a) and c) respectively. Right: Images after averaging over 8 cardiac cycles.

The MI on the left ventricle (LV) is easily detectable, as shown in Figure 1. Images from single heartbeats are noisier compared to those that are averaged over heartbeats, though the infarct is still visible and well-delineated.

## Conclusions

The high rates of sub-sampling enabled by through-time radial GRAPPA make DCE imaging with improved temporal resolution and spatial resolution possible, while maintaining contrast. Accelerated radial acquisitions have the potential to deliver single heart beat high-resolution DCE images without the need for breath-holds or complicated motion-correction methods.
